# Healthcare workers’ knowledge and attitudes towards sterilization and reuse of medical devices in primary and secondary care public hospitals in Nepal: A multi-centre cross-sectional survey

**DOI:** 10.1371/journal.pone.0272248

**Published:** 2022-08-01

**Authors:** Gopal Panta, Ann K. Richardson, Ian C. Shaw, Patricia A. Coope

**Affiliations:** 1 School of Health Sciences, University of Canterbury, Christchurch, New Zealand; 2 School of Physical and Chemical Sciences, University of Canterbury, Christchurch, New Zealand; 3 College of Education, Health and Human Development, University of Canterbury, Christchurch, New Zealand; Pi Research Consultancy Center, BANGLADESH

## Abstract

**Background:**

Healthcare facilities reprocess and sterilize reusable medical devices before each invasive clinical procedure, such as surgery, to prevent person-to-person or environmental transmission of pathogens through medical devices. We conducted a nationwide multi-centre cross-sectional survey in primary and secondary-care public hospitals in Nepal to assess the knowledge and attitudes of healthcare workers towards sterilization and reuse of medical devices.

**Methods:**

We carried out a multi-centre cross-sectional survey comprising eleven primary-care (two district-level and nine district hospitals) and two secondary-care (zonal hospitals) public hospitals which covered all seven provinces of Nepal. Survey questionnaires were distributed to 234 healthcare workers including doctors, nurses, paramedics, and office assistants (involved in medical device reprocessing); 219 (93.6%) returned the completed questionnaire. Descriptive analyses of demographic information, knowledge and attitude responses of survey participants were performed. Logistic regression and ordinal regression models for complex samples were used to investigate associations between responses and independent variables.

**Results:**

Except for a few areas, more than 70% of healthcare workers had proper knowledge about different aspects of sterilization and reuse of medical devices. Paramedics and office assistants were less likely to have the correct knowledge in different aspects compared to nurses. Permanent staff were more likely to give correct answers to some knowledge questions compared to temporary staff. Previous infection control training was positively associated with correct responses to some knowledge items. Most of the healthcare workers had positive attitudes towards different aspects of sterilization and reuse of medical devices, and nurses were more likely to have positive attitudes compared with other staff categories.

**Conclusions:**

Most of the healthcare workers had correct knowledge and positive attitudes towards most areas of sterilization and reuse of medical devices. However, they need proper education and training in some areas such as sterilization procedures, storage of sterilized devices, prion decontamination and standard precautions.

## Background

Reusable medical devices used for invasive clinical procedures (e.g. surgery) are reprocessed and sterilized before each use to prevent person-to-person or environmental transmission of pathogens to patients and healthcare workers. Inadequate reprocessing and sterilization of such devices in healthcare facilities leads to an increased risk of device-associated infections [1]. It has been estimated that 10.2% (CI_95_: 9.0% - 13.0%) of hospitalized patients acquire healthcare-associated infections (HAIs) in developing countries—surgical site infections (SSIs) are the most reported HAIs in these countries [2]. HAIs can prolong a patient’s stay in the hospital, cause long-term disability, increase the financial burden for health systems, increase costs for patients and their families, and result in deaths [2]. Studies in Nepal have also shown a higher proportion of SSIs (2.7 to 23.0 per 100 patients) in patients who had undergone different surgeries in different tertiary-care hospitals [3–6]. The studies have indicated that inadequate reprocessing and sterilization of reusable medical devices could be one of the factors contributing to the higher rates of SSIs. We previously reported a high proportion of steam sterilization failures in primary and secondary care public hospitals in Nepal and poor compliance with the recommended practices for medical device reprocessing and sterilization [7, 8].

Appropriate knowledge among healthcare workers about different aspects of sterilization and reuse of medical devices is a basic requirement for ensuring adequate sterilization of reusable medical devices. Studies in different countries have shown that a substantial proportion of health workers do not have adequate knowledge of some disinfection and sterilization issues [9–12]. Some studies have investigated the attitudes of healthcare workers towards elements of infection control in healthcare facilities [13, 14]. However, specific documentation about knowledge and attitudes of healthcare workers in Nepal towards sterilization and reuse of medical devices could not be found.

We report findings of a cross-sectional survey assessing knowledge and attitudes of healthcare workers towards sterilization and reuse of medical devices in primary (district-level and district hospitals) and secondary (zonal hospitals) care public hospitals in Nepal.

## Methods

### Survey questionnaire

A survey questionnaire consisting of items related to knowledge and attitudes of healthcare workers towards sterilization and reuse of medical devices was developed and used. The questionnaire had three sections, including demographic information of survey participants, a section with knowledge-related items, and a section covering attitude-related items. The knowledge and attitude sections contained categorical response items, rating scale items and some open-ended questions. The rating scales had a minimum value of one and a maximum value of seven. Some of the rating scale items in both knowledge and attitude sections were deliberately worded negatively to minimize the “acquiescent response bias” [15].

To develop the questionnaire, a literature search was conducted in online databases including Google Scholar, Medline and CINAHL to identify studies focusing on knowledge and attitudes of healthcare workers towards sterilization and reuse of medical devices in different countries. Based on the obtained studies [9, 11, 16–26] and some national/international guidelines and standards [1, 27–31] of the related field, a draft questionnaire was developed. The draft questionnaire was further reviewed by experts in Nepal and New Zealand [a public health expert from Nepal, a professor and physician working in the area of infection prevention and control in Nepal, a clinical nurse specialist working in infection prevention and control in a tertiary care hospital in New Zealand, supervisors of this study (AKR and ICS) and a biostatistician (PAC)] and revised after receiving feedback from the experts. The revised questionnaire was translated into Nepali by the researcher (GP). The translated items were added to the main questionnaire; therefore, the questionnaire included items in both English and Nepali. The questionnaire was field-tested in one of the district hospitals in Nepal. After considering the feedback obtained from the respondents and the experiences gained in the field, the questionnaire was further modified and finalized. Finally, the questionnaire was also reviewed by the Human Ethics Committee of the University of Canterbury and the Nepal Health Research Council (NHRC).

### Sample design and sample size

This study was part of a comprehensive study, which was designed to estimate the steam sterilization failure proportion in primary and secondary care public hospitals in Nepal, assess compliance of these hospitals with standard practices for reprocessing and steam sterilization of medical devices, and understand healthcare workers’ knowledge and attitudes towards sterilization and reuse of medical devices. The number of hospitals included in the study was primarily determined for estimating the steam sterilization failure proportion—this has been reported in detail elsewhere [7]. There were 88 primary and secondary care public hospitals which were categorized into three types–district-level (16), district (62) and zonal (10) hospitals [32]. Of those, a stratified cluster random sample of 13 hospitals was selected for the study– 2 district-level, 9 districts and 2 zonal hospitals. Most districts in the country have one district hospital, which is the first line of service outlet providing hospital-level care in the district. These hospitals provide healthcare services including inpatient, outpatient, dental, general surgical, child health and emergency services. District-level hospitals are satellite primary-care hospitals that are smaller than district hospitals in terms of services, catchment area and infrastructure; these hospitals are located in a few districts only. The services provided by these hospitals include inpatient, outpatient and minor surgical services. Zonal Hospitals provide specialized services equivalent to secondary-level care. Such specialized services are related to paediatrics, gynaecology, general surgery, general medicine, eye care, dermatology, orthopaedics and psychiatry. Family planning, immunisation, antenatal services, delivery services and laboratory services were provided by the hospitals in all categories.

It was expected that the distribution of the responses to rating scale items (a minimum value of 1 and a maximum value of 7) in the survey questionnaire would be skewed and so its shape was approximated by a right-angled triangle. To estimate the mean response for any scale item with a margin of error of 0.3 and 95% level of confidence, the sample size was determined to be 85 healthcare workers at minimum.

### Sample selection

The selection of hospitals within each hospital type was random. For district hospitals, we wanted to have the selected hospitals spread across the seven provinces, so the hospitals were chosen following a systematic random sampling method. The selection of the hospitals has been discussed in detail elsewhere [7].

It was required to ensure that staff from each category including doctors, nurses, paramedics (health assistants and auxiliary health workers) and office assistants (only those involved in medical device reprocessing and sterilization) from each hospital received the survey questionnaire. The number of healthcare workers belonging to some categories, such as doctors, was very small making simple random sampling practically impossible within a hospital. Therefore, the questionnaires were distributed to as many healthcare workers as possible. Careful consideration was taken to avoid the biased distribution of the survey questionnaire among healthcare staff. Office assistants not involved in medical device reprocessing activities and interns were excluded from the study.

### Data collection procedure

A survey questionnaire (S1 Questionnaire) was provided to each healthcare worker participating in the study. The researcher explained to each participant about the items in the questionnaire, the rating scales, and their interpretations. The participants were asked to return the survey questionnaires to the researcher in person immediately after completion. Some healthcare workers (e.g., office assistants) were unable to complete the questionnaire by themselves because of poor or no literacy. For them, they were interviewed by the researcher to complete a questionnaire on behalf of each participant. Altogether, 234 healthcare workers in thirteen primary and secondary care hospitals in Nepal received the survey questionnaires from 5 June to 16 December 2016.

### Data management and analysis

A unique number was assigned to each hospital and recorded on each survey questionnaire used in the study. Information from the completed questionnaires was entered in a database (Excel spreadsheet) every day. After the completion of fieldwork, data in the spreadsheets were imported into IBM SPSS Statistics 24 software. Imported data sets were checked for errors and discrepancies. Identified errors and discrepancies were then corrected by referring to the completed questionnaires. To make analysis and interpretation clearer, responses to negatively worded questions [i.e. a response of 7 (strongly agree) in the rating scale indicated an incorrect response] in the original questionnaire were recorded in the reverse order to ensure that all responses of 7 (strongly agree) indicated correct responses.

Descriptive analysis of demographic information of survey participants and responses to knowledge and attitude items was performed. The analysis included but was not limited to, calculation of proportions, assessing associations between variables, and some regression analyses. Ordinal Regression Models were used to analyze the association of responses to questions in rating scale formats with different variables related to healthcare workers, including duration of healthcare work, type of healthcare profession, prior infection control training, healthcare employment status (permanent or contract) and practice of autoclave operation. An association between variables was considered statistically significant if P value ≤ 0.05. The complex sample design was taken into account when analyzing the data.

### Ethical consideration

Ethical clearance was obtained from the Human Ethics Committee of the University of Canterbury (HEC 2015/139). In addition, approval was obtained from the Nepal Health Research Council (13/2016). Written consent was obtained from the medical superintendent or official in charge of each of the thirteen selected hospitals before initiating research activities in the hospitals. Written consents were also obtained from all healthcare workers participating in the survey. Written information about the study was provided to all the medical superintendents or the officials in charge and the participants of the survey before obtaining the written consent.

## Results

### Characteristics of the healthcare workers participating in the study

A total of 234 healthcare workers were provided with the survey questionnaire out of which 291 (93.6%) returned the completed questionnaire. Participants from zonal hospitals were 59 (26.9%), 138 (63.0%) were from district hospitals and 22 (10.1%) were from district-level hospitals. The number of female participants (63.9%) was higher than that of male participants (36.1%). The age of the healthcare workers participating in the survey ranged from 18 to 59 years. More than 55% of the participants were aged ≤ 30 years. The study participants had from 2 months to 39 years (mean = 9.7 years, SD = 9.7) of work experience in healthcare. Nurses comprised the highest proportion and office assistants comprised the lowest proportion (Table 1). The proportion of survey participants reporting prior training in infection control/prevention was 52.0% (n = 114) and the proportion of participants reporting having operated an autoclave at some time was 42.0% (n = 92).

**Table 1:** Characteristics of healthcare workers participating in the survey.

Characteristics	Number (Percentage)
**Gender**	
Male	79 (36.1)
Female	140 (63.9)
**Age**	
20 or under	7 (3.2)
21–30	116 (53.0)
31–40	56 (25.6)
41–50	21 (9.6)
51–60	18 (8.2)
Age missing	1 (0.5)
**Duration of work in healthcare**	
≤ 1 year	37 (16.9)
> 1 to ≤ 5 years	60 (27.4)
> 5 to ≤ 10 years	45 (20.6)
> 10 years	75 (34.2)
Unknown	2 (0.9)
**Profession**	
Doctors	47 (21.5)
Nurses	117 (53.4)
Paramedics	38 (17.3)
Office assistants*	17 (7.8)
**Healthcare employment status**	
Permanent	124 (56.6)
Temporary (contract)	95 (43.4)
**Training in infection control**	
Yes	114 (52.0)
No	104 (47.5)
**Practice of autoclave operation**	
Yes	92 (42.0)
No	127 (58.0)
**Total**	**219 (100.0)**

* their level of education ranges from illiteracy to a maximum of year 10 (class 10) of school education

### Knowledge of healthcare workers

#### Responses to questions in rating scale formats

The majority of the responses to the rating-scale knowledge questions were towards the correct (strongly agree) end (Table 2). Ordinal regression models showed that there was a significant statistical association between the responses to all these knowledge questions and one or more variables, including duration of healthcare work, type of healthcare profession, infection control training, and employment status (Table 3). However, none of the responses was significantly associated (p>0.05) with the self-reported practice of autoclave operation by healthcare workers.

**Table 2:** Healthcare workers’ responses to five knowledge questions in rating scale format.

Knowledge sentence	Percentage of healthcare workers marking a number in the rating scale (%)*						
	1	2	3	4	5	6	7
**K1**	5.4	0.5	0.0	1.5	0.8	4.9	86.8
**K2**	7.0	0.8	0.0	2.6	2.5	7.4	79.6
**K3** **	22.2	6.3	5.4	13.9	1.5	4.3	46.6
**K4** **	11.3	0.8	0.6	6.8	2.0	5.0	73.5
**K5**	36.5	4.8	2.9	7.7	4.7	6.0	37.4

* The level of participant’s agreement with the sentence gradually increases from 1 to 7, 1 = Strongly disagree, 4 = Neither agree nor disagree, 7 = Strongly agree; 2 and 3 indicate some level of disagreement but less than strong disagreement, 5 and 6 indicate some level of agreement but less than strong agreement

** Statement was negatively worded in the original questionnaire distributed to the healthcare workers

**K1**: Used medical devices harbour a variety of microorganisms that could be transmitted among patients and healthcare workers.

**K2**: Sterilization kills all microorganisms including spores.

**K3**: Immersion of medical devices in 2% glutaraldehyde for 10 minutes does not constitute sterilization.

**K4**: Autoclaving is more effective than chemical methods for killing microorganisms.

**K5**: Wet sterilized packs of medical devices obtained from autoclaving are considered to be contaminated.

**Table 3:** Complex samples—ordinal regression models for responses of healthcare workers to knowledge questions in rating-scale formats.

Predictor variable	Odds Ratio	95% Confidence Interval	P value***
***Model 1*:** *Used medical devices harbour a variety of microorganisms that could be transmitted among patients and healthcare workers*			
Duration of healthcare work*	1.06	0.99–1.12	0.07
Healthcare profession			
Doctors	0.78	0.20–2.94	0.68
Paramedics	0.35	0.16–0.77	**0.01**
Office Assistants	1.76	0.24–12.56	0.54
Nurses**	1.00		
Infection control training	0.76	0.45–1.29	0.28
Healthcare employment status			
Permanent	1.78	1.01–3.15	**0.047**
Temporary (contract)**	1.00		
Practice of autoclave operation	1.09	0.44–2.68	0.83
***Model 2*:** *Sterilization kills all microorganisms including spores*			
Duration of healthcare work*	1.02	0.99–1.04	0.08
Healthcare profession			
Doctors	0.68	0.29–1.56	0.33
Paramedics	0.29	0.07–1.15	0.07
Office Assistants	1.44	0.23–8.83	0.66
Nurses**	1.00		
Infection control training	2.12	1.02–4.42	**0.046**
Healthcare employment status			
Permanent	1.04	0.53–2.02	0.90
Temporary (contract)**	1.00		
Practice of autoclave operation	0.83	0.41–1.67	0.57
***Model 3*:** *Immersion of medical devices in 2% glutaraldehyde for 10 minutes does not constitute sterilization*.			
Duration of healthcare work*	0.97	0.93–1.00	0.054
Healthcare profession			
Doctors	0.20	0.12–0.34	**<0.001**
Paramedics	0.25	0.12–0.50	**0.001**
Office Assistants	0.12	0.03–0.45	**0.005**
Nurses**	1.00		
Infection control training	1.64	0.96–2.79	0.07
Healthcare employment status			
Permanent	2.02	1.23–3.31	**0.01**
Temporary (contract)**	1.00		
Practice of autoclave operation	0.64	0.39–1.02	0.06
***Model 4*:** *Autoclaving is more effective than chemical methods for killing microorganisms*.			
Duration of healthcare work*	0.93	0.89–0.97	**0.003**
Healthcare profession			
Doctors	0.52	0.20–1.36	0.16
Paramedics	0.34	0.12–0.96	**0.04**
Office Assistants	0.32	0.17–0.58	**0.002**
Nurses**	1.00		
Infection control training	2.64	1.19–5.86	**0.02**
Healthcare employment status			
Permanent	2.42	1.30–4.50	**0.01**
Temporary (contract)**	1.00		
Practice of autoclave operation	0.64	0.27–1.51	0.28
***Model 5*:** *Wet sterilized packs of medical devices obtained from autoclaving are considered to be contaminated*.			
Duration of healthcare work*	1.03	1.01–1.05	**0.01**
Healthcare profession			
Doctors	0.41	0.14–1.17	0.09
Paramedics	0.33	0.17–0.65	**0.001**
Office Assistants	1.50	0.49–4.58	0.43
Nurses**	1.00		
Infection control training	1.37	0.71–2.61	0.31
Healthcare employment status			
Permanent	0.64	0.30–1.36	0.22
Temporary (contract)**	1.00		
Practice of autoclave operation	1.25	0.63–2.50	0.48

* Continuous variable

** Reference category

*** Statistically significant results are shown in bold

### Temperature and time for autoclaving

Of the healthcare workers, 80.0% (CI_95_: 75.4–84.0) specified 121°C as the recommended temperature for steam sterilization for the autoclaves used at their hospitals, whereas 54.6% (CI_95_: 43.8–64.9) of the healthcare workers reported 30 minutes as the effective holding/exposure period for sterilizing wrapped medical devices (Table 4). There was no statistically significant correlation between stated sterilization temperature and holding period (r = 0.03, p = 0.56). A logistic regression model for complex samples showed that infection control training and healthcare profession were statistically significantly associated with the knowledge of correct sterilization temperature, i.e. 121°C (Table 5). All the office assistants (involved in medical device reprocessing including operation of autoclaves) mentioned ‘Don’t know’ in the space provided for writing the required temperature for sterilizing medical devices.

**Table 4:** Temperature and holding period of autoclave cycles as stated by the respondents.

	Estimate Percentage	95% Confidence Interval
**Temperature (**°C)		
121	80.0%	75.4% - 84.0%
<121	11.9%	7.9% - 17.7%
>121	2.4%	0.9% - 6.2%
Don’t know	5.7%	3.6% - 8.9%
**Holding period (mins)**		
30	54.6%	43.8% - 64.9%
<30	40.5%	31.1% - 50.7%
>30	4.9%	2.1% - 11.0%

**Table 5:** Complex samples—logistic regression model for knowledge of recommended temperature.

Predictor Variable	Odds Ratio	95% Confidence Interval	P value***
***Model*:** *For autoclaves being used in this hospital*, *the temperature inside the autoclave chamber while sterilizing medical devices is 121°C*.			
Duration of healthcare work*	1.00	0.93–1.07	0.97
Healthcare profession			
Doctors	0.51	0.19–1.32	0.15
Paramedics	0.25	0.09–0.66	**0.01**
Office Assistants	0.03	0.00–0.18	**0.002**
Nurses**	1.00		
Infection control training	3.16	1.62–6.20	**0.003**
Healthcare employment status			
Permanent	1.54	0.50–4.78	0.42
Temporary (contract)**	1.00		
Practice of autoclave operation	0.63	0.17–2.23	0.43

* Continuous variable

** Reference category

*** Statistically significant results are shown in bold

#### Shelf-life of sterilized medical devices

About 17.8% (CI_95_: 12.2–25.2) of the health workers thought that sterilized, wrapped medical devices can be stored for less than 7 days at room temperature before using them, and 78.8% (CI_95_: 69.4–85.9) thought that they can be stored for 7 days. Only 3.4% (CI_95_: 0.7–15.2) of the healthcare workers thought that sterilized, wrapped medical devices could be stored for more than 7 days before use.

#### Decontamination of specific medical devices

The proportions of healthcare workers able to correctly identify the single highest level of decontamination process appropriate for auroscope ear-pieces, ear syringes and thermometers were 41.1% (CI_95_: 32.5–50.3), 29.4% (CI_95_: 21.0–39.6) and 32.7% (CI_95_: 23.7–43.1) respectively. Similarly, the proportions of healthcare workers able to correctly identify the single highest level of decontamination process appropriate for metal forceps, scalpel handles and vaginal specula were 91.3% (CI_95_: 85.2–95.0), 84.7% (CI_95_: 79.4–88.9) and 87.9% (CI_95_: 78.6–93.5) respectively (Table 6). Of the healthcare workers, 45.2% (CI_95_: 36.2–50.3) thought that the routine sterilization process for medical devices needed to be changed for neurosurgical procedures and only one of them (a doctor) indicated that prions are resistant to routine sterilization processes. Indeed, 6.8% (CI_95_: 3.7–12.0) of the healthcare workers wrote ‘Don’t know’ leaving the yes/no options unchecked.

**Table 6:** Participants’ opinion on the highest level of decontamination appropriate for reusable medical devices.

Medical device	The appropriate highest-level decontamination process		
	Cleaning	Disinfection	Sterilization
Auroscope earpiece	39.3% (CI_95_: 29.7–49.8)	41.1%* (CI_95_: 32.5–50.3)	19.6% (CI_95_: 15.0–25.2)
Ear syringe	26.7% (CI_95_: 18.4–36.9)	43.9% (CI_95_: 35.0–53.3)	29.4%* (CI_95_: 21.0–39.6)
Metal forceps	1.2% (CI_95_: 0.5–2.8)	7.5% (CI_95_: 4.4–12.6)	91.3%* (CI_95_: 85.2–95.0)
Scalpel handle	5.2% (CI_95_: 2.1–12.2)	10.1% (CI_95_: 5.8–17.0)	84.7%* (CI_95_: 79.4–88.9)
Thermometer	66.8% (CI_95_: 56.5–75.8)	32.7%* (CI_95_: 23.7–43.1)	0.5% (CI_95_: 0.1–3.8)
Vaginal speculum	0.9% (CI_95_: 0.2–3.9)	11.3% (CI_95_: 5.7–21.2)	87.9%* (CI_95_: 78.6–93.5)

* Recommended decontamination process

### Attitudes of healthcare workers

Most of the responses to the attitude questions were towards the positive (strongly agree) side (Table 7). An Ordinal Regression Model for complex samples (Table 8) showed that attitudes towards policies and standards were statistically significantly associated with the healthcare profession when adjusted for other variables, including duration of work in healthcare, infection control training, current employment status and practice of autoclave operation of the healthcare workers. Similarly, attitudes towards training were significantly associated with the healthcare profession and prior infection control/prevention training (Table 9). Attitudes towards cleaning were statistically significantly associated with current employment status and healthcare profession (Table 10). Healthcare workers’ feeling of safety while being treated as a patient using medical devices sterilized in their hospitals was statistically significantly associated with the healthcare profession (Table 11). Healthcare workers’ opinion about considering all patients as potentially HIV positive was statistically significantly associated with the healthcare profession and prior infection control/prevention training adjusted for other variables (Table 12). Similarly, employment status was statistically significantly associated with the agreement that deviation from routine reprocessing procedures for medical devices is not required when the devices had been used in patients with HIV (Table 12). An Ordinal Regression Model for complex samples showed that the responses to questions related to patient safety (A1), risk of infection (A2), availability of sterilizers and supplies (A4), monitoring (A5), and the number of staff involved in medical device reprocessing (A10) were not statistically significantly associated with any of the variables mentioned above.

**Table 7:** Healthcare workers’ responses to twelve attitude questions.

Attitude sentence	Percentage of healthcare workers marking a number in the rating scale (%)*						
	1	2	3	4	5	6	7
**A1**	4.6	0.7	1.9	5.2	3.4	2.3	81.9
**A2**	2.3	1.0	0.4	1.2	1.4	6.3	87.5
**A3** **	10.7	1.4	1.9	5.1	2.0	7.7	71.1
**A4**	3.7	0.0	1.1	0.5	3.3	10.8	80.7
**A5** **	3.0	1.3	0.5	3.1	0.4	3.5	88.2
**A6**	1.1	0.5	0.0	0.7	2.2	6.6	89.0
**A7** **	12.8	0.5	1.2	1.3	0.7	3.9	79.6
**A8** **	5.6	4.3	1.0	3.9	2.4	3.0	79.8
**A9**	4.9	4.0	3.9	4.6	6.8	7.7	68.1
**A10** **	46.1	6.9	9.1	10.1	4.8	7.0	16.1
**A11**	20.1	1.1	1.5	4.9	2.9	6.2	63.3
**A12** **	48.3	7.7	2.9	5.6	0.5	4.8	30.1

* The level of participant’s agreement with the sentence gradually increases from 1 to 7, 1 = Strongly disagree, 4 = Neither agree nor disagree, 7 = Strongly agree; 2 and 3 indicate some level of disagreement but less than strong disagreement, 5 and 6 indicate some level of agreement but less than strong agreement

** Statement was negatively worded in the original questionnaire distributed to the healthcare workers

**A1**: Reuse of medical devices is an important patient safety issue.

**A2**: Decontamination of medical devices reduces the risk of infection in patients and healthcare workers.

**A3**: Written policies and standards are necessary for ensuring appropriate decontamination of medical devices.

**A4**: Availability of sterilizers and supplies supports routine decontamination of medical devices.

**A5**: Monitoring of the sterilization process deserves the same attention to detail applied to other key patient care activities.

**A6**: Training on the operation of sterilizer/autoclave helps ensure adequate sterilization of medical devices.

**A7**: Cleaning before sterilization is a necessary process.

**A8**: If an instrument is not soiled visibly, we still need to clean it before sterilization.

**A9**: I would feel safe being treated as a patient using medical devices sterilized in this hospital.

**A10**: The number of staff involved in the decontamination of medical devices in this hospital is adequate.

**A11**: Every patient attending healthcare facilities must be considered potentially HIV positive.

**A12**: Deviation from routine reprocessing procedures for medical devices is not required when the devices had been used in patients with HIV.

**Table 8:** Complex samples—ordinal regression model for the attitude of healthcare workers towards policies and standards.

Predictor Variable	Odds Ratio	95% Confidence Interval	P value***
***Model*:** *Written policies and standards are necessary for ensuring appropriate decontamination of medical devices*.			
Duration of healthcare work*	1.0	0.93–1.01	0.09
Healthcare profession			
Doctors	0.5	0.21–1.06	0.07
Paramedics	0.6	0.24–1.49	0.24
Office Assistants	0.3	0.15–0.83	**0.02**
Nurses**	1.0		
Infection control training	1.2	0.46–2.92	0.73
Healthcare employment status			
Permanent	1.1	0.54–2.18	0.80
Temporary (contract)**	1.0		
Practice of autoclave operation	0.8	0.43–1.57	0.52

* Continuous variable

** Reference category

*** Statistically significant results are shown in bold

**Table 9:** Complex samples—ordinal regression model for the attitude of healthcare workers towards training.

Predictor Variable	Odds Ratio	95% Confidence Interval	P value***
***Model*:** *Training on the operation of sterilizer/autoclave helps ensure adequate sterilization of medical devices*			
Duration of healthcare work*	1.05	1.00–1.10	0.05
Healthcare profession			
Doctors	0.32	0.13–0.82	**0.02**
Paramedics	0.82	0.13–5.03	0.81
Office Assistants^**1**^	1.34	1.00–18.46	0.81
Nurses**	1.00		
Infection control training	0.31	0.15–0.73	**0.01**
Healthcare employment status			
Permanent	0.37	0.12–1.16	0.08
Temporary (contract)**	1.00		
Practice of autoclave operation	1.251	0.46–3.38	0.626

* Continuous variable

** Reference category

*** Statistically significant results are shown in bold

^1^ all of the office assistants strongly agreed with this statement (i.e. marked 7 on the rating scale) but the response of one of the office assistants was assumed to be 6 instead of 7 to make the odds ratio estimable.

**Table 10:** Complex samples—ordinal regression models for the attitude of healthcare workers towards cleaning of medical devices.

Predictor Variable	Odds Ratio	95% Confidence Interval	P value***
***Model*:** *If an instrument is not soiled visibly*, *we still need to clean it before sterilization*			
Duration of healthcare work*	0.98	0.92–1.05	0.53
Healthcare profession			
Doctors	0.42	0.12–1.42	0.14
Paramedics	0.24	0.06–0.89	**0.04**
Office Assistants	0.49	0.14–1.68	0.23
Nurses**	1.00		
Infection control training	1.02	0.41–2.52	0.96
Healthcare employment status			
Permanent	1.40	1.06–1.84	**0.02**
Temporary (contract)**	1.00		
Practice of autoclave operation	1.00	0.45–2.23	1.00

* Continuous variable

** Reference category

*** Statistically significant results are shown in bold

**Table 11:** Complex samples—ordinal regression model for the attitude of healthcare workers towards being treated as a patient.

Predictor Variable	Odds Ratio	95% Confidence Interval	P value***
***Model*:** *I would feel safe being treated as a patient using medical devices sterilized in this hospital*			
Duration of healthcare work*	0.99	0.96–1.03	0.68
Healthcare profession			
Doctors	0.23	0.06–0.87	**0.03**
Paramedics	1.32	0.23–7.76	0.73
Office Assistants	2.84	0.34–23.36	0.30
Nurses**	1.00		
Infection control training	1.49	0.68–3.26	0.28
Healthcare employment status			
Permanent	0.95	0.40–2.27	0.90
Temporary (contract)**	1.00		
Practice of autoclave operation	1.11	0.26–4.72	0.88

* Continuous variable

** Reference category

*** Statistically significant results are shown in bold

**Table 12:** Complex samples—ordinal regression models for the attitude of healthcare workers towards HIV and reprocessing of medical devices.

Predictor Variable	Odds Ratio	95% Confidence Interval	P value***
***Model 1*:** *Every patient attending healthcare facilities must be considered potentially HIV positive*			
Duration of healthcare work*	1.00	0.96–1.04	0.93
Healthcare profession			
Doctors	0.68	0.31–1.48	0.29
Paramedics	0.37	0.16–0.84	**0.02**
Office Assistants	0.43	0.11–1.72	0.21
Nurses**	1.00		
Infection control training	2.58	1.29–5.15	**0.01**
Healthcare employment status			
Permanent	1.35	0.74–2.46	0.29
Temporary (contract)**	1.00		
Practice of autoclave operation	0.52	0.24–1.12	0.09
***Model 2*:** *Deviation from routine reprocessing procedures for medical devices is not required when the devices had been used in patients with HIV*			
Duration of healthcare work*	0.95	0.93–0.98	**< 0.01**
Healthcare profession			
Doctors	0.74	0.35–1.57	0.39
Paramedics	1.02	0.42–2.46	0.96
Office Assistants	0.71	0.30–1.71	0.41
Nurses**	1.00		
Infection control training	1.48	0.83–2.63	0.16
Healthcare employment status			
Permanent	3.12	2.13–4.56	**< 0.01**
Temporary (contract)**	1.00		
Practice of autoclave operation	1.55	0.73–3.29	0.23

* Continuous variable

** Reference category

*** Statistically significant results are shown in bold

## Discussion

We obtained a response rate of 93.6% in this survey and this rate was similar to those obtained in other relevant studies which involved the administration of questionnaires to participants in person [33, 34]. We found that more than 70% of healthcare workers had good knowledge of different aspects of sterilization and reuse of medical devices except for chemical (glutaraldehyde) sterilization and wet sterilized packages, for which less than 50% of healthcare workers strongly agreed with the knowledge statements. Regression models revealed that paramedics and office assistants, compared to nurses, were less likely to have correct knowledge about different issues of sterilization and reuse of medical devices (see Tables 3 and 5). Better knowledge among nurses could have been because of their greater involvement in routine infection control activities in hospitals [35]. Compared to temporary staff, permanent staff were more likely to give correct answers to knowledge questions related to microbial contamination of used medical devices, glutaraldehyde sterilization, and the effectiveness of autoclaving. This could be because of better exposures and educational opportunities given to permanent staff than to temporary staff. Previous infection control training was positively associated with correct responses to some aspects of knowledge, including those related to microbial contamination of reused medical devices, the effectiveness of autoclaving, and steam sterilization temperature. The practice of autoclave operation was not statistically significantly associated with responses to any of the knowledge questions. When the healthcare workers were asked whether they ever operated an autoclave, they were likely to answer ‘yes’ even if they had operated an autoclave only once. Therefore, the reported practice of autoclave operation could not have been statistically significantly associated with the responses to any of the knowledge questions.

Most of the healthcare workers (80.0%) in primary and secondary care hospitals correctly specified the temperature (i.e. 121°C) recommended for steam sterilization of medical devices. However, only 54.7% of them could identify the correct holding period for wrapped medical devices (i.e. 30 min) which is stated in the national Reference Manual for Infection Prevention and Healthcare Waste Management [30]. In principle, higher sterilization temperatures require a shorter holding period [36]. However, there was no significant correlation between the temperature and the holding period reported by the healthcare workers. This indicates a gap in knowledge among healthcare staff about the appropriate temperature and holding period for steam sterilization. Knowledge among healthcare workers about appropriate decontamination of some medical devices, including metal forceps, scalpel handles and vaginal specula (91.3%, 84.7% and 87.9% respectively) was relatively superior compared to knowledge about appropriate decontamination of some other medical devices such as auroscope ear-pieces, ear syringes and thermometers (41.1%, 29.4% and 32.7% respectively). Though 45.2% of the healthcare workers thought that routine sterilization processes for medical devices needed to be changed for neurosurgical procedures, only one knew about prions. This indicates a knowledge gap among healthcare workers about prions and their resistance to routine sterilization processes.

The proportion of healthcare workers reporting prior training on infection control/prevention in this study (51.6%) was higher than the proportion reported by a previous study (27.1%) conducted in 2006 in Kathmandu, the capital city of Nepal [33]. This difference might be due to gradual improvement in the frequency of training activities over the intervening period. In this study, 22.2% of healthcare workers strongly agreed that immersion of medical devices in 2% glutaraldehyde for 10 minutes results in sterilization, 46.6% of the healthcare workers strongly disagreed with the statement and about 14% of them remained neutral. In three similar UK studies, 13%, 16% and 27% of healthcare workers thought that soaking in 2% glutaraldehyde for 10 minutes led to sterilization [9, 11, 12]. In light of these previous findings, the response of healthcare workers in Nepal was not surprising. However, the findings of this study indicate that there is confusion among healthcare workers regarding this matter. Glutaraldehyde (2%) is usually used for high-level disinfection of medical devices that cannot withstand high temperatures. Immersion of medical devices to 2% glutaraldehyde solution for a longer time is commonly considered as ‘sterilization’ [1, 30, 37]. Two UK studies assessed the knowledge of healthcare workers about appropriate decontamination of specific medical devices [11, 12]. These two previous studies reported that percentages of healthcare workers identifying the appropriate decontamination process were 68.9% and 72.0% for auroscope ear-pieces, 22.7% and 11.2% for ear syringes, 100.0% and 96.9% for metal forceps, 91.2% and 90.3% for scalpel handles, 72.7% and 77.7% for thermometers, and 93.3% and 97.8% for vaginal specula. The results of our study were comparable with the results from these studies, even though our study was conducted more than 15 years later. However, comparatively lower percentages of healthcare workers in Nepal were able to correctly identify appropriate decontamination processes for auroscope ear-pieces (41.1%) and thermometers (32.7%). In all three studies (including our study), fewer than 30% of healthcare workers correctly identified the appropriate decontamination process for ear syringes. These similarities between the findings of our study and previous studies for different aspects of sterilization and reuse of medical devices were obtained despite differences in contexts, geographical locations, study time, study participants and the structure of the questions in the questionnaire. These similarities corroborate our findings.

Though, in general, the attitudes of healthcare workers towards different aspects of sterilization and reuse of medical devices were found to be positive, nurses were more likely to have positive attitudes towards different aspects of sterilization and reuse of medical devices compared with doctors, paramedics and office assistants. The attitudes of healthcare workers towards policies and standards in our study were found similar to the findings of a study conducted by Sukhlecha *et al*. (2015) in a tertiary hospital in western India [38] They reported that 84.3% of healthcare workers (including final-year students and interns, nurses, laboratory technicians and sanitary staff) strongly agreed or agreed that sterilization guidelines/policy in their hospital were useful. In our study, 80.8% of healthcare workers in primary and secondary hospitals indicated positive attitudes (5, 6 or 7 on a 7-points rating scale) towards written policies and standards about decontamination of medical devices. Of the healthcare workers participating in this survey, 63.3% strongly agreed that every patient attending healthcare facilities must be considered potentially HIV positive. This finding agreed with the findings of previous studies from Mexico and Iran, where 60.0% of dentists responded ‘of course’ to the statement, and 90.3% of dentists agreed with the statement respectively [24, 26].

### Implications of the study

In this study, we report that all office assistants (support staff) answered ‘don’t know’ when asked for the temperature used for steam sterilization of medical devices in their hospitals. It is noteworthy that all of them were involved in medical device reprocessing activities, including steam sterilization in their hospitals. Their level of education ranges from illiteracy to a maximum of year 10 (class 10) of school education. Interestingly, we previously reported that they were involved in 97.0% (CI_95_: 87.5% - 99.3%) of the steam sterilization cycles [8] and 71.0% (CI_95_: 46.8% - 87.2%) of these cycles failed to sterilize medical devices when tested with biological indicators [7]. In addition, compliance with standard practices for medical device reprocessing and steam sterilization was very poor in these hospitals [8]. One of the key reasons behind such poor compliance might be the low education level of staff responsible for medical device reprocessing. Therefore, for an improvement in the current situation of medical device reprocessing and sterilization, staff with better educational qualifications (e.g., higher secondary level) or existing paramedic or nursing staff with adequate training should be utilized for medical device reprocessing and sterilization. Though all categories of healthcare staff need continued training and education, our study revealed that paramedics need more attention to improve their knowledge in this area compared with nurses.

We found a positive association between prior infection prevention/control training and correct or positive responses to many knowledge and attitude questions, and no statistically significant negative association was found between training and responses except for one attitude question related to healthcare workers’ feeling of safety. These findings support the importance of training in improving the knowledge of healthcare workers regarding the sterilization of medical devices. ‘Infection prevention and healthcare waste management is the only training program related to infection prevention/control in Nepal. This provides some information on medical device reprocessing and sterilization for healthcare workers; however, the training time allocated to this is only three hours [39]. We previously reported poor compliance in primary and secondary public hospitals in Nepal with the standard practices for medical device reprocessing and sterilization [8]; such poor compliance might be because of inadequate staff training. Medical device reprocessing and sterilization is a specialized area comprising several scientific processes, and specific training on this topic can be useful for providing adequate knowledge and skills for healthcare workers. Indeed, in some countries, staff involved in the sterilization of medical devices are required to have specific certification in sterilizing technology; for example, in New Zealand, certification in sterilization technology with at least 400 hours of study is required [40].

This study shows that healthcare workers in Nepal need to be properly educated about some important issues related to medical device sterilization and reuse. One such issue is prion decontamination. Prions are primarily found in central nervous system tissue and can cause one or a group of fatal degenerative brain diseases known as transmissible spongiform encephalopathies (TSEs; e.g, Creutzfeldt-Jakob disease (CJD)). Prions are resistant to conventional physical and chemical methods of disinfection and sterilization [41]. Only one participant (a doctor) mentioned prions when participants were asked if routine sterilization procedures needed to be changed for medical devices used for neurosurgical procedures. Though we did not find any literature reporting cases of TSEs in Nepal (this might be because TSEs are not diagnosed and/or recorded in Nepal), the possibility of their occurrence cannot be ruled out. Cases of CJD have been documented in the northern part of neighbouring India [42, 43]. Contaminated neurosurgical instruments have been identified as a source of prions for a small proportion of reported cases of iatrogenic CJD globally [44]. None of the hospitals included in the study were performing neurosurgical procedures, but there are higher-level public and private hospitals in Nepal which perform neurosurgical procedures. The finding that very few healthcare workers in primary and secondary care hospitals knew about prions may be relevant to the surgery-related risk profiles of higher-level hospitals. There are recommendations for modifying routine procedures for decontamination of medical devices likely to be contaminated with prions [45] and there is a need to educate healthcare staff in Nepal (especially those working in higher-level hospitals) about prions and related decontamination procedures. Our study also indicated the need for educating healthcare workers in appropriate decontamination of specific medical devices, such as auroscope ear-pieces, ear syringes and thermometers. Similarly, the study also identified the need for educating staff on the temperature and time required for steam sterilization and chemical sterilization procedures such as glutaraldehyde sterilization.

About 79% of the healthcare workers in primary and secondary care hospitals in Nepal thought that wrapped sterilized medical devices could be stored for seven days at room temperature before use. A shelf-life of 7 days is very much less than the shelf-life indicated by many previous studies, and there is be growing support for the event-based determination of shelf-life rather than the time-based shelf-life of sterilized medical devices [46–49]. When event-based shelf-life is implemented, wrapped medical devices are stored for a longer time, i.e. until an event, such as tearing or damage to the wrapping, leads to possible microbial contamination. Implementing a short shelf-life for sterilized packages of medical devices demands additional resources because of the need for more frequent sterilization. In a resource-limited country like Nepal, it might be more economical to implement a longer shelf-life for sterilized packages. At the same time, the importance of appropriate sterilization, packaging (material and method), storage, environmental conditions, and handling of the packages cannot be overlooked [50]. There is no universal recommendation for the shelf-life of sterilized packages. Lakhan et al. (2013) conducted a review of evidence about the shelf-life of sterilized packaged items and pointed out the need for risk assessment before implementing event-based or time-based shelf-life for sterilized packages [51]. When considering the shelf-life of sterilized packages of medical devices, dryness is important because moisture provides a vector for microbial access. Guidelines advise that wet sterilized packages of medical devices should be considered contaminated since wet packages facilitate access and growth of microorganisms [1, 37]. However, knowledge of healthcare workers about this was found to be divided, with 37.4% of the healthcare workers strongly agreeing that wet packages should be considered contaminated while a similar percentage (36.5%) strongly disagreed. Paramedics and newer healthcare workers need more education about this than other healthcare workers.

In this study, more than 48% of the healthcare workers strongly agreed that deviation from routine reprocessing procedures for medical devices is required when the devices had been used in patients with HIV. This opinion is against the principles of universal/standard precautions for all patient care [52–54]: medical devices used for HIV-positive patients do not need to be reprocessed differently. This opinion could be a manifestation of HIV-related stigma and discrimination in Nepal. Similar manifestations of stigma were reported by some other studies; for example, 97.2% of healthcare workers in rural north India agreed that it is necessary to take extra infection control precautions for patients with HIV [25, 55, 56]. These findings emphasize the importance of healthcare worker education in standard precautions and HIV transmission.

### Strengths and limitations

A complex sample design (stratification of hospitals and clustering of participants) was considered when analyzing data from this study, and therefore, the findings of this study can be expected to represent all primary and secondary care public hospitals in Nepal. This study includes different categories of healthcare workers (doctors, nurses, paramedics, office assistants) and broadly covers their knowledge and attitudes towards different issues related to sterilization and reuse of medical devices. We not only investigated the knowledge and attitudes of healthcare workers but also identified some factors likely to be associated with the knowledge and attitudes of healthcare workers through regression models for complex samples. The findings of this study may be useful in formulating interventions to improve sterilization and reuse of medical devices in hospitals in Nepal and may contribute to ensuring patient safety in these hospitals.

One of the limitations of this study was that tertiary-care public hospitals, private hospitals, and small healthcare facilities (e.g., primary health centres) were not included in this study. However, the findings of this study are likely relevant to these healthcare facilities because of similarities in contexts. Another limitation was the approach used for the selection of healthcare workers for the survey. The selection of healthcare workers was not random for practical reasons, and to ensure the inclusion of smaller workforce groups. Survey questionnaires were provided to as many healthcare workers as possible. This could have led to the biased enrolment of more approachable healthcare workers.

## Conclusion

This study reports the knowledge and attitudes of healthcare workers towards different aspects of sterilization and reuse of medical devices in primary and secondary care public hospitals in Nepal. Though most of the healthcare workers had correct knowledge and positive attitudes towards most areas of sterilization and reuse of medical devices, this study identifies areas where healthcare workers in Nepal need better education and training. This may help policymakers, public health managers and infection prevention professionals develop and implement interventions to ensure adequate sterilization and reuse of medical devices; this will likely contribute to improved patient safety in the hospitals in Nepal. We also identified issues relating to medical device reprocessing (e.g., prion decontamination, chemical sterilization and shelf-life of sterilized packages) that need further exploration and improvement in Nepal. The findings of this study may also be useful for other low- and middle-income countries to facilitate improvement in sterilization and reuse of medical devices in their healthcare facilities.

## Supporting information

S1 Questionnaire Knowledge and attitude of healthcare workers towards sterilization and reuse of medical devices. (DOCX)
