# Supplementary material for: Healthcare workers’ knowledge and attitudes towards sterilization and reuse of medical devices in primary and secondary care public hospitals in Nepal: A multi-centre cross-sectional survey
Source: PLoS One. 2022 Aug 1;17(8):e0272248. doi: 10.1371/journal.pone.0272248 (PMC9342727; doi:10.1371/journal.pone.0272248)
Supplement: S1 Questionnaire — (DOCX) [file pone.0272248.s001.docx]

1. **DEMOGRAPHIC INFORMATION**

Please check (√) in the box that corresponds to your answer.

1. Gender: ☐Male ☐Female ☐Other
2. Age (in years): _____________
3. What is your highest level of medical or health education?

☐PhD

☐Masters (MD/MS or Equivalent) ☐Masters (MN/MSc Nursing or Equivalent)

☐Bachelors (MBBS or Equivalent) ☐Bachelors (BN/BSc Nursing or Equivalent)

☐Certificate (Health Assistant/HA) ☐Certificate (Staff Nurse)

☐Auxiliary Health Worker (AHW) ☐Auxiliary Nurse Midwife (ANM)

☐ Other (please specify) ______________________________________________

1. Your Job Title: _____________________
2. For how long have you been working as a healthcare worker? ______________ years
3. Your current employment status is ☐Permanent ☐Contract /Temporary
4. **KNOWLEDGE**
5. Have you ever received training on Infection Control /Prevention

☐Yes ☐No

**To answer the following questions, please circle the number on the scale to show how you agree with the statement.**

1. Used medical devices harbour a variety of microorganisms that could be transmitted among patients and healthcare workers.

| 1 | 2 | 3 | 4 | 5 | 6 | 7 |
| --- | --- | --- | --- | --- | --- | --- |
| Strongly Disagree | | Neither Agree  or Disagree | | | Strongly Agree | |

1. Sterilization kills all microorganisms including spores.

| 1 | 2 | 3 | 4 | 5 | 6 | 7 |
| --- | --- | --- | --- | --- | --- | --- |
| Strongly Disagree | | Neither Agree  or Disagree | | | Strongly Agree | |

1. Immersion of medical devices in 2 % glutaraldehyde for 10 minutes constitutes sterilisation.

| 1 | 2 | 3 | 4 | 5 | 6 | 7 |
| --- | --- | --- | --- | --- | --- | --- |
| Strongly Disagree | | Neither Agree  or Disagree | | | Strongly Agree | |

1. Autoclaving is not as effective as chemical methods for killing microorganisms.

| 1 | 2 | 3 | 4 | 5 | 6 | 7 |
| --- | --- | --- | --- | --- | --- | --- |
| Strongly Disagree | | Neither Agree  or Disagree | | | Strongly Agree | |

1. Wet sterilized packs of medical devices obtained from autoclaving are considered to be contaminated.

| 1 | 2 | 3 | 4 | 5 | 6 | 7 |
| --- | --- | --- | --- | --- | --- | --- |
| Strongly Disagree | | Neither Agree  or Disagree | | | Strongly Agree | |

1. For autoclaves being used at your hospital, the temperature inside the autoclave chamber while sterilizing medical devices is

______°C

1. For how long should wrapped medical devices be kept at **this temperature** (mentioned in the answer to question 7) to sterilize them?

___________­­­_ minutes

1. How long can we store wrapped sterilized medical devices at room temperature before using them?

_____________ days

1. Do you ever operate an autoclave? ☐Yes ☐No
2. Please check (√)the **single** highest level of decontamination process appropriate for the following medical devices
3. Auroscope ear piece ☐*Cleaning* ☐*Disinfection* ☐*Sterilization*
4. Ear syringe ☐*Cleaning* ☐*Disinfection* ☐*Sterilization*
5. Metal forceps ☐*Cleaning* ☐*Disinfection* ☐*Sterilization*
6. Scalpel handle ☐*Cleaning* ☐*Disinfection* ☐*Sterilization*
7. Thermometer ☐*Cleaning* ☐*Disinfection* ☐*Sterilization*
8. Vaginal speculum ☐*Cleaning* ☐*Disinfection* ☐*Sterilization*
9. Do we need to change the routine sterilization process for medical devices for neurosurgical procedures?

☐Yes ☐No

If yes, why?

___________________________________________________________________________

____________________________________________________________________________________________________________________________________________

1. **ATTITUDE**

**To answer the following questions, please circle the number on the scale to show how you agree with the statement.**

1. Reuse of medical devices is an important patient safety issue.

| 1 | 2 | 3 | 4 | 5 | 6 | 7 |
| --- | --- | --- | --- | --- | --- | --- |
| Strongly Disagree | | Neither Agree  or Disagree | | | Strongly Agree | |

1. Decontamination of medical devices reduces the risk of infection in patients and healthcare workers.

| 1 | 2 | 3 | 4 | 5 | 6 | 7 |
| --- | --- | --- | --- | --- | --- | --- |
| Strongly Disagree | | Neither Agree  or Disagree | | | Strongly Agree | |

1. Written policies and standards are not necessary for ensuring appropriate decontamination of medical devices.

| 1 | 2 | 3 | 4 | 5 | 6 | 7 |
| --- | --- | --- | --- | --- | --- | --- |
| Strongly Disagree | | Neither Agree  or Disagree | | | Strongly Agree | |

1. Availability of sterilizers and supplies supports routine decontamination of medical devices.

| 1 | 2 | 3 | 4 | 5 | 6 | 7 |
| --- | --- | --- | --- | --- | --- | --- |
| Strongly Disagree | | Neither Agree  or Disagree | | | Strongly Agree | |

1. Monitoring of the sterilization process does not deserve the same attention to detail applied to other key patient care activities.

| 1 | 2 | 3 | 4 | 5 | 6 | 7 |
| --- | --- | --- | --- | --- | --- | --- |
| Strongly Disagree | | Neither Agree  or Disagree | | | Strongly Agree | |

1. Training on the operation of sterilizer/autoclave helps ensure adequate sterilization of medical devices.

| 1 | 2 | 3 | 4 | 5 | 6 | 7 |
| --- | --- | --- | --- | --- | --- | --- |
| Strongly Disagree | | Neither Agree  or Disagree | | | Strongly Agree | |

1. Cleaning before sterilization is an unnecessary process.

| 1 | 2 | 3 | 4 | 5 | 6 | 7 |
| --- | --- | --- | --- | --- | --- | --- |
| Strongly Disagree | | Neither Agree  or Disagree | | | Strongly Agree | |

1. If an instrument is not soiled visibly, we do not need to clean it before sterilization.

| 1 | 2 | 3 | 4 | 5 | 6 | 7 |
| --- | --- | --- | --- | --- | --- | --- |
| Strongly Disagree | | Neither Agree  or Disagree | | | Strongly Agree | |

1. I would feel safe being treated as a patient using medical devices sterilized in this hospital.

| 1 | 2 | 3 | 4 | 5 | 6 | 7 |
| --- | --- | --- | --- | --- | --- | --- |
| Strongly Disagree | | Neither Agree  or Disagree | | | Strongly Agree | |

1. The number of staff involved in decontamination of medical devices in this hospital is not adequate.

| 1 | 2 | 3 | 4 | 5 | 6 | 7 |
| --- | --- | --- | --- | --- | --- | --- |
| Strongly Disagree | | Neither Agree  or Disagree | | | Strongly Agree | |

1. Every patient attending healthcare facilities must be considered potentially HIV positive.

| 1 | 2 | 3 | 4 | 5 | 6 | 7 |
| --- | --- | --- | --- | --- | --- | --- |
| Strongly Disagree | | Neither Agree  or Disagree | | | Strongly Agree | |

1. Deviation from routine reprocessing procedures for medical devices is required when the devices had been used in patients with HIV.

| 1 | 2 | 3 | 4 | 5 | 6 | 7 |
| --- | --- | --- | --- | --- | --- | --- |
| Strongly Disagree | | Neither Agree  or Disagree | | | Strongly Agree | |
